# COVID-19 and Risk of Acute Ischemic Stroke and Acute Lung Injury in Patients With Type II Diabetes Mellitus: The Anti-inflammatory Role of Metformin

**DOI:** 10.3389/fmed.2021.644295

**Published:** 2021-02-19

**Authors:** Hayder M. Al-kuraishy, Ali I. Al-Gareeb, M. Alblihed, Natália Cruz-Martins, Gaber El-Saber Batiha

**Affiliations:** ^1^Department of Clinical Pharmacology and Medicine, College of Medicine, Al-Mustansiriyia University, Baghdad, Iraq; ^2^Department of Microbiology, College of Medicine, Taif University, Taif, Saudi Arabia; ^3^Faculty of Medicine, University of Porto, Porto, Portugal; ^4^Institute for Research and Innovation in Health (i3S), University of Porto, Porto, Portugal; ^5^Laboratory of Neuropsychophysiology, Faculty of Psychology and Education Sciences, University of Porto, Porto, Portugal; ^6^Department of Pharmacology and Therapeutics, Faculty of Veterinary Medicine, Damanhour University, Damanhour, Egypt

**Keywords:** COVID-19, acute ischaemic stroke, diabates mellitus, metformin, SARS-CoV-2

## Abstract

**Background:** Coronavirus disease 19 (COVID-19) is regarded as an independent risk factor for acute ischemic stroke (AIS) due to the induction of endothelial dysfunction, coagulopathy, cytokine storm, and plaque instability.

**Method:** In this retrospective cohort study, a total of 42 COVID-19 patients with type 2 diabetes mellitus (T2DM) who presented with AIS within 1 week of displaying COVID-19 symptoms were recruited. According to the current anti-DM pharmacotherapy, patients were divided into two groups: a Metformin group of T2DM patients with COVID-19 and AIS on metformin therapy (850 mg, 3 times daily (*n* = 22), and a Non-metformin group of T2DM patients with COVID-19 and AIS under another anti-DM pharmacotherapy like glibenclamide and pioglitazone (*n* = 20). Anthropometric, biochemical, and radiological data were evaluated.

**Results:** Ferritin serum level was lower in metformin-treated patients compared to non-metformin treated patients (365.93 ± 17.41 vs. 475.92 ± 22.78 ng/mL, *p* = 0.0001). CRP, LDH, and D-dimer serum levels were also lowered in metformin-treated patients compared to non-metformin treated patients (*p* = 0.0001). In addition, lung CT scan scores of COVID-19 patients was 30.62 ± 10.64 for metformin and 36.31 ± 5.03 for non-metformin treated patients.

**Conclusion:** Metformin therapy in T2DM patients was linked to a lower risk of AIS during COVID-19. Further studies are needed to observe the link between AIS in COVID-19 diabetic patients and metformin therapy.

## Introduction

Coronavirus disease 19 (COVID-19) is a worldwide pandemic, caused by the severe acute respiratory syndrome-coronavirus 2 (SARS-CoV-2), that began in December 2019. Among the multiple mechanisms of virus action, the ability of the spike protein to bind to angiotensin converting enzyme 2 (ACE2) is the most prominent one, being around 10 times higher than the equivalent SARS-CoV (1). The ACE2 receptors involved in viral entry are highly expressed in different tissues, mainly in lung pneumocyte type II cells. The interaction between SARS-CoV-2 and ACE2 leads to a down-regulation of the protective ACE2 with the induction of hyper-inflammation and oxidative stress, and subsequent development of acute lung injury (ALI) and acute respiratory distress syndrome (ARDS) (2). Also, a reduction of ACE2, which is involved in the metabolism of angiotensin II (AngII), leads to vasoconstriction, hypertension, coagulopathy, and inflammatory reactions that together increase the risk of acute ischemic stroke (AIS) (3). Belani et al. (4) found that COVID-19 is regarded as an independent risk factor for AIS due to the induction of endothelial dysfunction, coagulopathy, cytokine storm, and plaque instability. A systemic review and meta-summary by Tan et al. (5) illustrated that high levels of D-dimer and inflammatory cytokines with the existence of anti-phospholipid antibodies seem to be linked to AIS in COVID-19 patients. On the other hand, the metabolic disturbances associated with type II diabetes mellitus (T2DM) may raise the risk of AIS during SARS-CoV-2 infection. This is because both T2DM and COVID-19 are linked to platelet activation, coagulation disorders, endothelial dysfunction, and insulin resistance (IR) that mutually contribute to the pathogenesis of AIS (6, 7). Also, Lee et al. (8) confirmed that glucose variability is associated with stroke severity and infarct volume in T2DM and non-DM patients. COVID-19 progression is accompanied with glucose variability due to the induction of IR and/or pancreatic injury by hypercytokinemia (9).

Metformin is a biguanide anti-diabetic agent used as a first-line drug in T2DM management, with anti-inflammatory and antioxidant properties (10). As its main actions, metformin increases ACE2 expression, thereby reducing the deleterious effect of high AngII in patients with cardiometabolic disorders and in the experimental model of ALI (11). A preliminary prospective study by Gao et al. (12) found that metformin therapy in COVID-19 patients with T2DM led to a raise in COVID-19 severity through potentiation of SARS-CoV-2 entry due to ACE2 receptors' overexpression. Likewise, ACE2 receptors improve neuronal functions and have neuroprotective activity, being down-regulated in AIS (13).

As a consequence, the rational of the present study was supported by the fact that the anti-inflammatory and antioxidant effects of metformin may improve the cardiometabolic profile in T2DM patients and COVID-19 (14). Thus, this study was aimed to illustrate the potential and bidirectional effect of metformin on both AIS and ALI in T2DM patients with COVID-19.

## Materials and Methods

### Study Design

In this retrospective cohort study, a total of 42 COVID-19 patients with T2DM with ages ranging from 41 to 66 years (12 females and 30 males) who presented with AIS within 1 week of showing COVID-19 symptoms were recruited from a single institutional COVID-19 sector and compared to 21 matched healthy controls. All COVID-19 patients with AIS were diagnosed cooperatively by an internist and neurologist using a full medical record, physical and neurological examinations, as well as biochemical and serological investigations. According to the current anti-diabetic pharmacotherapy, the patients were divided into two groups: Group I (the Metformin group), comprising T2DM patients with COVID-19 and AIS on metformin therapy (850 mg, 3 times daily) (*n* = 22), and Group II (the Non-metformin group), comprising T2DM patients with COVID-19 and AIS on another anti-diabetic pharmacotherapy (*n* = 20).

AIS patients were selected according to the diagnostic criteria of the American Academy of Neurology (15). The diagnosis of COVID-19 was done according to the COVID-19 diagnostic criteria reported by Ma et al. (16). All study procedures were done according to the Helsinki Declaration.

Recruited patients and healthy controls gave informed consent for their contribution to this study. This study was done in the Department of Clinical Pharmacology and Therapeutic and was approved by the Clinical Research and Ethical Committee Board, College of Medicine, Al-Mustansiryia University, Iraq, Bagdad, reference number MTR 21/3/2020.

### Inclusion and Exclusion Criteria

In this study, inclusion and exclusion criteria were defined as follows. T2DM patients aged > 40 years who presented with COVID-19 and AIS with/without metformin therapy were included in this study. History of cigarette smoking was considered in the inclusion criteria. On the other hand, patients with hemorrhagic stroke, T1DM, thyroid disorders, ischemic heart disease, valvular heart diseases, acute and chronic liver disorders, acute and chronic renal disorders, bacterial sepsis, mental and psychiatric disorders, complicated stroke, connective tissue disorders, or malignancies were excluded.

### Radiological Imaging

Brain computed tomography (CT) scan was done to confirm the focal neurological injury within 48 h from admission of AIS patients. Also, chest X-ray and lung CT scan investigations were done to confirm the existence of bilateral ground glass appearance in suspected COVID-19 patients. Lung CT scan scoring was done according to Francone et al. (17), where a score of 0: no lung involvement; score 1: <5% involvement of lung; score 2: involvement of 5–25% of lung; score 3: involvement of 26–50% of lung; score 4: involvement of 51–75% of lung; and score 5: involvement >75% of lung.

### Anthropometric Measurements

Body mass index (BMI) was measured by the specific equation: BMI = Weight (kg)/Height (m^2^). Systolic and diastolic blood pressure (SBP and DBP) were measured from the left arm at a supine position using an automated digital sphygmomanometer. Pulse pressure (PP) and mean arterial pressure (MAP) were estimated according to the Al-Kuraishy et al. (14) method.

### Biochemical Measurements

Following overnight fasting, 10 ml of venous blood sample was drawn for the assessment of inflammatory biomarkers and glycemic indices. Fasting blood glucose (FBG) and glycated hemoglobin (HbA1c) were measured by automated colorimetric assay. A homeostatic model for the assessment of insulin resistance (HOMA-IR) was used for determination of insulin resistance (IR). Lipid profile, including total triglyceride (TG), total cholesterol (TC) and high-density lipoprotein (HDL), were measured by the ELISA kit method. Other lipid profiles were measured indirectly by specific equations, including low density lipoprotein (LDL) by Friedewald formula, atherogenic index (AI) = log (TG/HDL), very-low-density lipoprotein (VLDL) = TG/5, atherogenic coefficient (AC) = TC-HDL/HDL, and cardiac risk ratio (CRR) = TC/HDL (18).

Serum ferritin (NV: 20–250 ng/ml), C-reactive protein (CRP) (NV: 0–5 mg/L), lactate dehydrogenase (LDH) (NV: 100–190 U/L), D-dimer (NV: <230 ng/ml), and serum insulin levels were measured by ELISA kit methods. Each sample was measured twice and a mean of the values was used to minimize measurement errors.

### Serological Investigations

A polymerase chain reaction anti-COVID-19 test to detect both immunoglobulin (Ig) M and IgG antibodies was used. The cut-off value was 0.00–0.04 mIu/ml for both IgM and IgG.

### Estimation of Stroke Risk Score

Stroke risk score (SRS) was estimated in AIS patients and healthy controls according to sheet questionnaires that considered age, BMI, blood pressure, and the presence of cardiometabolic disturbances according to the American Stroke Association (19).

### Statistical Analysis

Data analysis was made by using the Statistical Package for Social Sciences (SPSS) software (IBM Corp, V. 24; Armonk, NY, USA). Results were presented as mean ± standard deviation (SD), absolute values, and percentage. One-way analysis of variance (ANOVA) and unpaired *t* tests were used for the assessment of differences between groups. Pearson correlation was used for calculation of the correlations level. The level of significance was regarded as *p* value <0.05.

## Results

**Figure 1:**
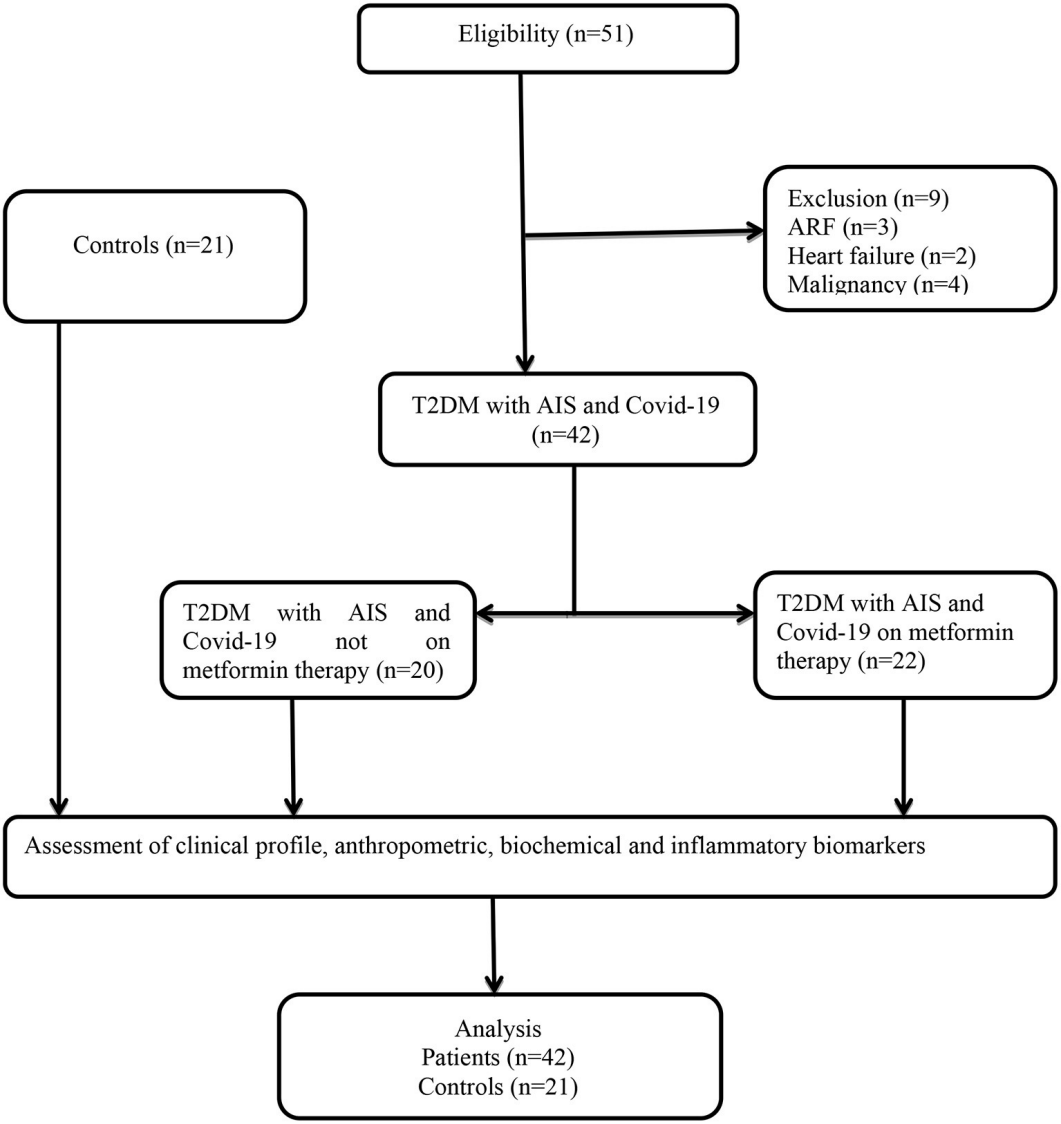
Consort-flow chart of the study.

In the present study, a total of 51 T2DM patients with COVID-19 and AIS were recruited; nine patients were excluded, three due to acute renal failure (ARF), two due to heart failure, and four due to malignancy. Thus, only 42 patients completed the study and were compared to 21 healthy controls. As stated above, patients were divided according to the diabetic pharmacotherapy into metformin-treated (*n* = 22), 52.38%, and non-metformin treated (*n* = 20), 47.61%, groups. Both groups of patients and healthy controls were investigated for the effect of metformin on clinical features and inflammatory and cardiometabolic profiles of T2DM patients with COVID-19 and AIS (Figure 1).

Regarding patients' demographic features (Table 1), there were no differences for both age and gender (*p* > 0.05). The positive family history for T2DM and AIS, as well as smoking status, was higher in AIS patients compared to controls (*p* = 0.001). Concerning the anti-diabetic pharmacotherapy, 52.28% of patients were on metformin therapy and 47.61% were on another diabetic pharmacotherapy, such as glibenclamide, pioglitazone, or sodium-glucose co-transporter (SGT2) inhibitors.

**Table 1:** Patients' demographic characteristics.

**Parameters**	**Healthy controls**	**Covid-19/AIS/T2DM patients**	***p* value**
*n*	21 (33.30)	42 (66.67)	0.01
Age (years)	48.08 ± 6.51	47.28 ± 6.73	0.65
Male gender	14 (66.67)	30 (71.42)	0.69
Positive family history	3 (14.28)	29 (69.04)	0.001
Smoking	5 (23.80)	31 (73.80)	0.001
**Co-morbidities**			
Hypertension		36 (85.71)	
IHD		22 (52.38)	
Dyslipidemia		31 (73.80)	
Previous AIS		11 (26.19)	
Duration of T2DM (years)		3.5 ± 1.21	
**Diabetes therapy**			
Metformin		22 (52.38)	
Non-metformin		20 (47.61)	
Glibenclamide		15 (75.00)	
Pioglitazone		2 (10.00)	
SGT2 inhibitors		3 (15.00)	
Another drug therapy			
Aspirin	8 (38.09)	19 (45.23)	0.59
Clopidogril		11 (26.19)	
Fenofibrate		6 (14.28)	
Statins		9 (21.42)	
β-blockers		31 (73.80)	
CCBs		18 (42.85)	
Antivirals		42 (100)	
Antibiotics		40 (95.23)	
Corticosteroids		20 (47.61)	
Oxygen support		19 (45.23)	
Enoxaparin			
At Covid-19		6 (14.28)	
At AIS		31 (73.80)	

*Data presented as n, %, mean ± SD. IHD, ischemic heart disease; AIS, acute ischemic stroke; CCB, calcium channel blocker*.

Regarding clinical presentation of T2DM patients with COVID-19, all patients (*n* = 42) presented with AIS, 22 (52.38%) of them were on metformin therapy, and 20 (47.61%) were on another therapeutic regimen. No clinical signs or symptoms significantly differed in T2DM patients with COVID-19 and AIS regarding metformin therapy (*p* > 0.05) with the exception of unilateral paralysis, which was higher in non-metformin (100%) compared to metformin treated patients (100% vs. 81.81%, *p* = 0.004) (Table 2).

**Table 2:** Clinical presentation of T2DM patients with COVID-19 and acute ischemic stroke.

**Clinical findings**	**Metformin**	**Non-metformin**	***p* value**
	**(*n* = 22)**	**(*n* = 20)**	
Unilateral paralysis	18 (81.81)	20 (100)	0.004
Single limb paralysis	5 (22.72)	3 (15.00)	0.45
Aphasia	9 (40.90)	8 (40.00)	0.94
Dysartharia	11 (50.00)	11 (55.00)	0.7
Dysphagia	4 (18.18)	6 (30.00)	0.31
Delirium	2 (9.09)	2 (10.00)	0.9
Convulsion	3 (13.63)	4 (20.00)	0.53
Visual loss	1 (4.54)	2 (10.00)	0.45
Coma	5 (22.72)	3 (15.00)	0.45
Fever	21 (95.45)	18 (90.00)	0.46
Dyspnea	11 (50.0)	11 (55.00)	0.7
Headache	16 (72.72)	18 (90.00)	0.07
Diarrhea	4 (18.18)	3 (15.00)	0.74
Sweating	19 (86.36)	17 (85.00)	0.88
Anosmia	14 (63.63)	17 (85.00)	0.05

*Data presented as n, %*.

Concerning the cardiometabolic profile and inflammatory biomarkers in T2DM patients with COVID-19 and AIS (Table 3), most parameters were higher in patients compared to controls (*p* < 0.001), although no significant differences in BMI and CRR were stated. In metformin-treated patients, BMI, blood pressure profile, glycemic indices, hemoglobin, and WBC indices did not differ from that stated in non-metformin treated patients (*p* > 0.05). On the other hand, lipid profile, atherogenic indices, and inflammatory cytokines were lower in metformin-treated patients as compared to non-metformin treated patients (*p* < 0.05). Ferritin serum levels were lower in metformin-treated when compared to non-metformin treated patients (365.93 ± 17.41 ng/mL vs. 475.92 ± 22.78 ng/mL, *p* = 0.0001). In addition, the serum CRP levels were lower in metformin-treated when compared to non-metformin treated patients (*p* = 0.04). Both LDH and D-dimer serum levels were lowered in metformin-treated patients compared to non-metformin treated patients (*p* = 0.0001). Platelet count was reduced in non-metformin treated patients compared to metformin treated patients (*p* = 0.0001).

**Table 3:** Cardio-metabolic profile and inflammatory biomarkers in T2DM patients with COVID-19 and AIS regarding metformin therapy compared with the controls.

**Variables**	**Controls**	**Metformin**	**Non-metformin**	**A**	**B**	**C**	**ANOVA**
	**(*n* = 21)**	**(*n* = 22)**	**(*n* = 20)**				
BMI (kg/m^2^)	31.68 ± 3.12	31.99 ± 3.71	32.93 ± 3.92	ns	ns	ns	0.51
SBP (mmHg)	123.64 ± 7.36	144.82 ± 9.31	148.89 ± 9.65	0.0001	0.0001	ns	0.0001
DBP (mmHg)	78.56 ± 5.32	87.08 ± 6.47	90.61 ± 7.41	0.0001	0.0001	ns	0.0001
PP (mmHg)	45.08 ± 3.78	57.74 ± 4.92	58.22 ± 5.83	0.0001	0.0001	ns	0.0001
MAP (mmHg)	93.59 ± 7.41	106.33 ± 8.61	110.04 ± 8.46	0.0001	0.0001	ns	0.0001
TC (mg/dL)	147.05 ± 11.68	186.63 ± 9.42	273.52 ± 11.81	0.0001	0.0001	0.0001	0.0001
TG (mg/dL)	144.85 ± 9.51	212.68 ± 13.93	285.42 ± 17.45	0.0001	0.0001	0.0001	0.0001
VLDL (mg/dL)	28.97 ± 3.88	42.53 ± 8.56	57.08 ± 9.47	0.0001	0.0001	0.0001	0.0001
HDL-c (mg/dL)	55.89 ± 4.97	49.22 ± 6.72	40.81 ± 8.22	0.0001	0.0001	0.03	0.0001
LDL-c (mg/dL)	62.20 ± 7.61	94.90 ± 9.52	175.60 ± 11.83	0.0001	0.0001	0.0001	0.0001
CRR	2.63 ± 1.03	3.79 ± 2.61	6.70 ± 4.82	ns	0.004	0.01	0.0004
AI	0.06 ± 0.002	0.27 ± 0.01	0.47 ± 0.02	0.0001	0.0001	0.0001	0.0001
AC	1.63 ± 0.86	2.7 ± 0.96	5.83 ± 1.85	0.02	0.0001	0.0001	0.0001
FBG (mg/dL)	89.63 ± 6.03	144.71 ± 8.91	145.64 ± 8.22	0.0001	0.0001	ns	0.003
HbA1c (%)	5.3 ± 0.53	6.7 ± 2.41	7.4 ± 2.82	0.04	0.01	ns	0.007
Serum insulin (mIU/L)	8.13 ± 2.11	17.52 ± 3.81	18.56 ± 3.25	0.01	0.01	ns	0.003
HOMA-IR	1.63 ± 0.86	6.92 ± 2.61	7.04 ± 3.61	0.003	0.003	ns	0.002
S. Ferritin (ng/mL)	90.51 ± 10.87	365.93 ± 17.41	475.92 ± 22.78	0.0001	0.0001	0.0001	0.0001
CRP (mg/L)	3.16 ± 1.08	43.85 ± 6.04	48.54 ± 6.13	0.0001	0.0001	0.04	0.0001
LDH (U/L)	133.71 ± 9.53	287.92 ± 13.84	299.63 ± 14.75	0.0001	0.0001	0.0001	0.0001
D-dimer (ng/mL)	21.92 ± 3.17	285.53 ± 14.94	307.84 ± 15.82	0.0001	0.0001	0.0001	0.0001
Oxygen saturation (%)	98.99 ± 1.11	94.86 ± 3.61	94.62 ± 3.79	0.0001	0.0001	ns	0.0001
WBC (10^3^/μL)	8.30 ± 2.16	16.63 ± 3.85	17.92 ± 3.44	0.0001	0.0001	ns	0.0001
Neutrophil %	66.53 ± 4.81	77.42 ± 8.21	78.66 ± 8.11	0.0001	0.0001	ns	0.0001
Lymphocyte %	34.86 ± 6.92	16.58 ± 5.49	14.87 ± 4.31	0.0001	0.0001	ns	0.0001
Hb (mg/dL)	13.59 ± 1.43	12.53 ± 1.22	12.74 ± 1.64	0.04	ns	ns	0.04
Platelets (10^3^/μL)	342.69 ± 12.94	262.43 ± 12.49	209.45 ± 11.82	0.0001	0.0001	0.0001	0.0001

*Data presented as mean ± SD. ns, not significant; A, metformin vs. control; B, non-metformin vs. control; C, metformin vs. non-metformin; BMI, body mass index; SBP, systolic blood pressure; DBP, diastolic blood pressure; PP, Pulse pressure; MAP, mean arterial pressure; TG, total triglyceride; TC, total cholesterol (TC); HDL, high density lipoprotein; LDL, low density lipoprotein; AI, atherogenic index; VLDL, very low density lipoprotein; AI, atherogenic coefficient; CRR, cardiac risk ratio; FBG, Fasting blood glucose; HbA1c, glycated hemoglobin; HOMA-IR, Homeostatic model for assessment of insulin resistance; LDH, lactate dehydrogenase*.

**Figure 2:**
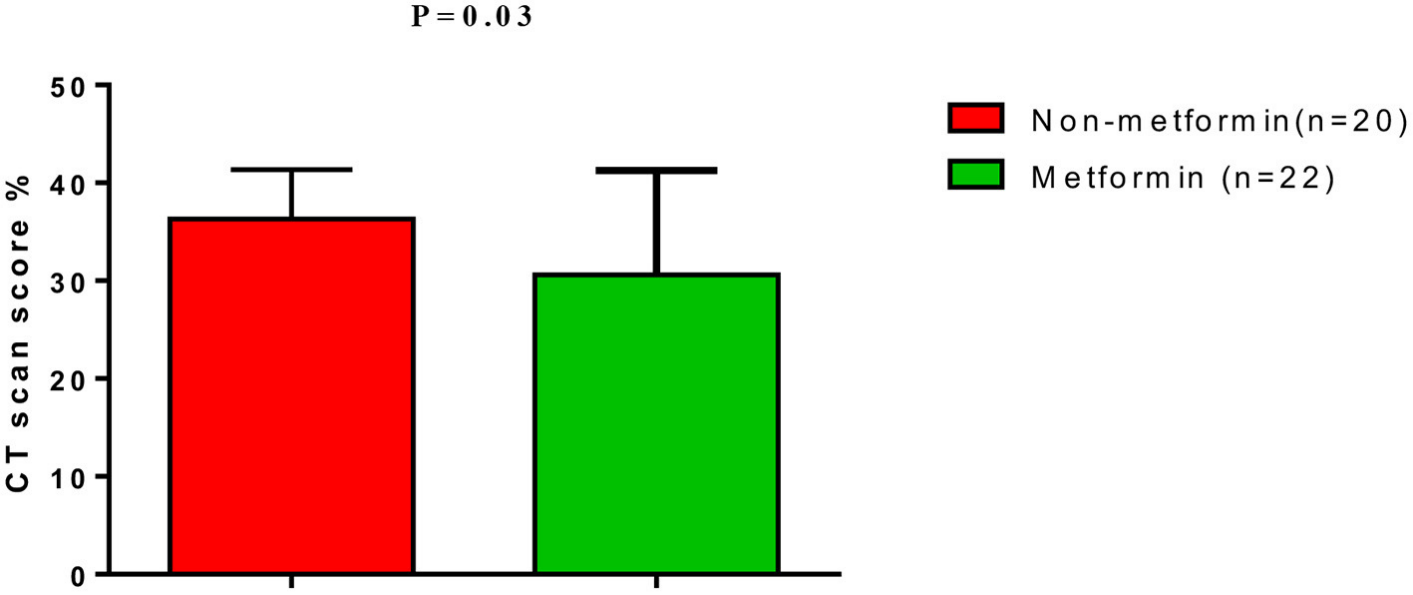
Lung CT scan score in T2DM patients with COVID-19 on metformin therapy.

**Figure 3:**
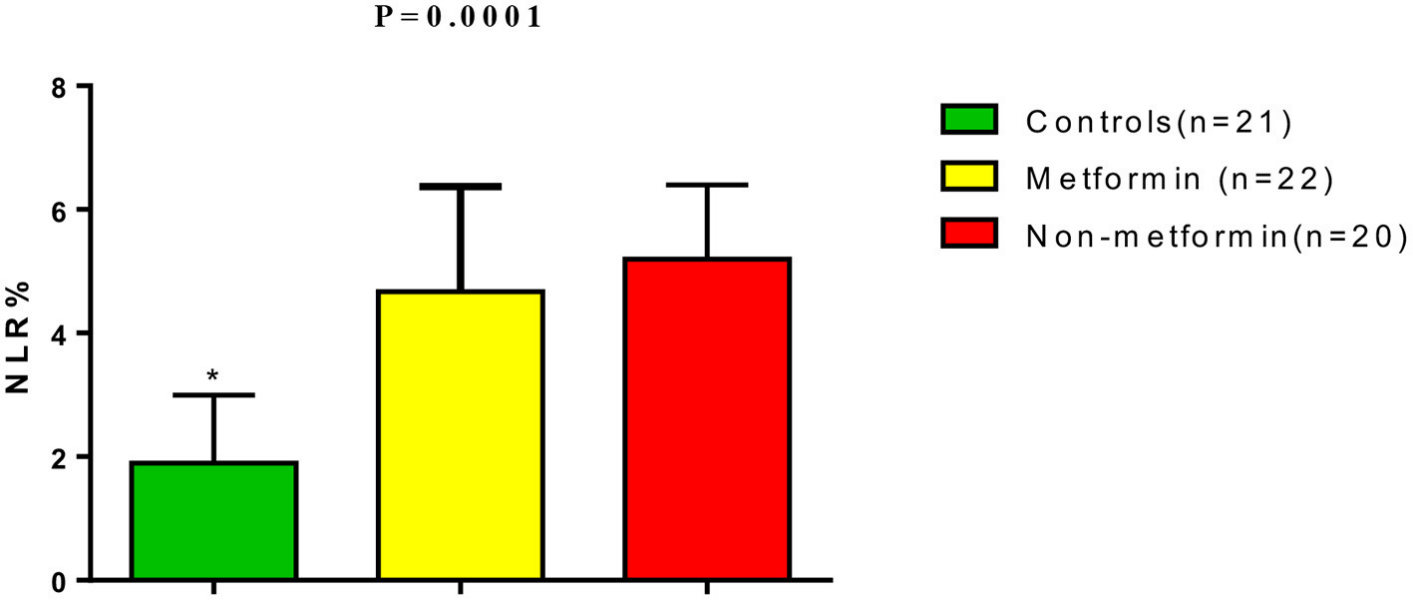
Neutrophil: lymphocyte ratio (NLR) in T2DM patients with COVID-19 and AIS compared with the controls. *P = 0.0001 compared to metformin and non-metformin treated patients.

In addition, lung CT scan score percentage of COVID-19 patients was 30.62 ± 10.64 for metformin-treated patients and 36.31 ± 5.03 for non-metformin treated patients (Figure 2). Moreover, the neutrophil: lymphocyte ratio (NLR) was higher in T2DM patients with COVID-19 and AIS compared to controls (*p* = 0.0001); however, NLR did not significantly differ between non-metformin treated and metformin-treated patients (5.2 ± 1.2 vs. 4.67 ± 1.7, *p* = 0.06) (Figure 3).

**Figure 4:**
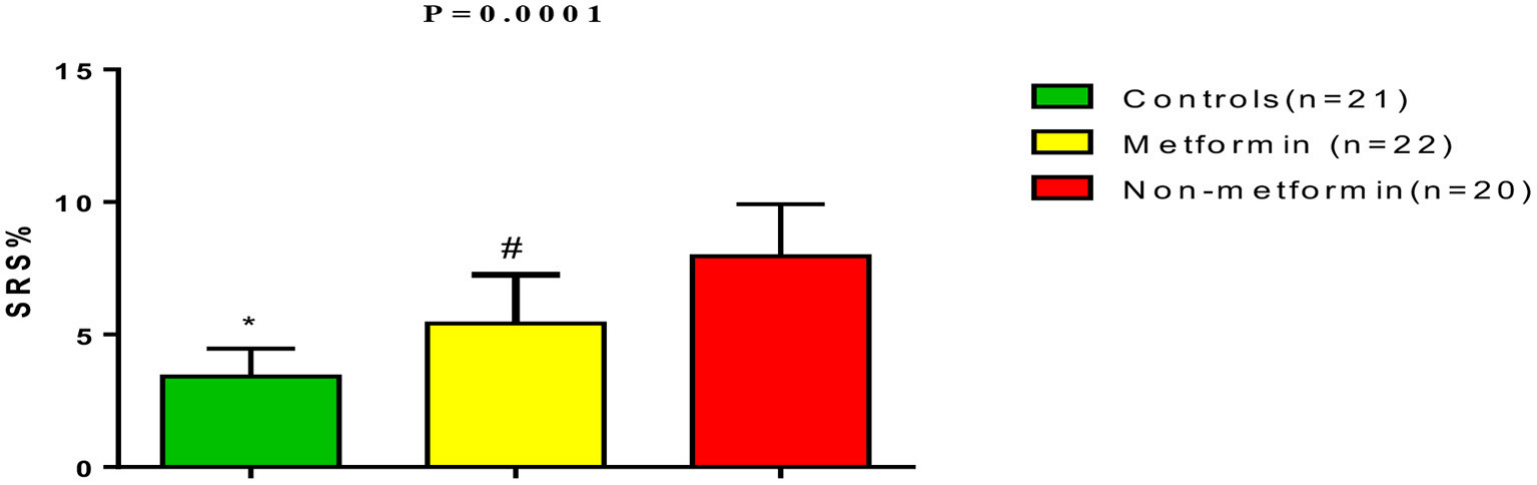
Stroke risk ratio (SRR) in T2DM patients with COVID-19 and acute ischemic stroke compared with the controls. *P = 0.0001 compared to metformin and non-metformin treated patients. ^#^*P* = 0.01 compared non-metformin treated patients.

Also, the SRR in T2DM patients with COVID-19 and AIS was higher in comparison to controls (*p* = 0.0001), although was lower in metformin-treated patients (3.42 ± 1.05) when compared to non-metformin treated patients (7.96 ± 1.96) (*p* = 0.01) (Figure 4). Also, SRR was correlated with the inflammatory biomarkers in T2DM patients with COVID-19 and AIS in both metformin-treated and non-metformin treated patients (*P* < 0.001) (Table 4).

**Table 4:** Correlation of inflammatory biomarkers in T2DM patients with COVID-19 and acute ischemic stroke.

**Biomarkers**	**Metformin (*****n*** **=** **22)**		**Non-metformin (*****n*** **=** **20)**	
	**r**	***p***	**r**	***p***
S. Ferritin (ng/mL)	0.56	0.006	0.77	0.0001
CRP (mg/L)	0.61	0.002	0.84	0.0001
LDH (U/L)	0.58	0.004	0.75	0.0001
D-dimer (ng/mL)	0.91	0.0001	0.96	0.0001
NLR%	0.55	0.005	0.56	0.006

*NLR, neutrophil: lymphocyte ratio; LDH, lactate dehydrogenase*.

## Discussion

In this study, it was illustrated that SARS-CoV-2 infection in T2DM patients augments the risk of AIS through the provocation of underlying cardio-metabolic disturbances, as reported by Zaki et al. (20). Most recruited patients had a positive family history of stroke, smoking, hypertension, T2DM, and dyslipidemia that increased the risk of AIS during the development of COVID-19, as confirmed by Saban-Ruiz et al. (21). Correspondingly, it has been shown that the risk of AIS during COVID-19 is approximately 5%, with 65.6% of AIS in COVDI-19 patients being labeled as cryptogenic stroke. A delay in the diagnostic investigation of COVID-19 patients may also contribute to the high rate of cryptogenic stroke (22).

In our retrospective cohort study, the clinical presentation of the disease started within 1 week after SARS-CoV-2 infection, as reported by Yaghi et al. (23) who stated in their retrospective cohort study a median of 10 days for AIS onset following COVID-19. COVID-19-induced-AIS may be due to a combination of cardio-metabolic disturbances and COVID-19-related mechanisms, such as hyper-coagulation, cytokine storm, vasculitis, endothelial dysfunction, and cardiomyopathy-induced cardiac arrhythmias (24). Notably, binding of SARS-CoV-2 to the ACE2 receptors, which are also abundant in the vascular endothelium, may trigger an inflammatory reaction and lymphocytic endothelitis with subsequent development of endothelial dysfunction and overall impairment of microcirculatory function (25). In addition, the endothelial dysfunction induced by SARS-CoV-2, along with the underlying T2DM-induced endothelial dysfunction, leads to cerebral microcirculatory failure with the development of brain ischemia and AIS (26). Thereby, the development of AIS in the T2DM patients of the present study might be due to a the pre-existence of cardio-metabolic disorders during the development of COVID-19 pneumonia.

On the other hand, data obtained in this study also underline the association between COVID-19 and elevation of inflammatory (CRP and ferritin), coagulation (D-dimer), and tissue injury biomarkers (LDH), consistent with recent published studies (27). Indeed, hyperinflammation and cytokine storm in COVID-19 increase the risk of multi-focal AIS due to impairment of vasodilator endothelial heparin sulfate, complement activation, and micro-vascular thrombosis (27). For example, a recent study comparing AIS alone with COVID-19-associated AIS showed no differences in cardio-metabolic risk factors, suggesting a specific mechanism of SARS-CoV-2 action in the pathogenesis of AIS (28). However, in the present study most recruited patients had a poor cardio-metabolic profile that predisposed them for the development of AIS. So, the pure mechanism of SARS-CoV-2-induced-AIS was not confirmed in our study due to limitations in the detection of SARS-CoV-2 at the infarction sites.

Indeed, SARS-CoV-2 binding to the ACE2 receptor in cardiomyocyte leads to myocardial injury with the induction of cardiac arrhythmia and increasing risk of thrombo-embolism-induced AIS (29). Respiratory insufficiency-induced hypoxemia and cytokine storm in COVID-19 also cause indirect cardiomyocyte injury (30). However, the echocardiographic profile and troponin serum levels were not addressed in this study.

Furthermore, in this study, 14.28 and 73.80% of recruited patients received enoxaparin at the time of COVID-19 and AIS diagnosis, respectively. Indeed, a delay in the initiation and the use of unstandardized enoxaparin therapy may increase both COVID-19 severity and complications, since enoxaparin therapy reduces the risk of thrombo-embolism and development of AIS (31). Also, enoxaparin improves the clinical outcomes of COVID-19 patients, as it exerts a remarkable anti-inflammatory and antiviral effect that attenuates COVID-19 coagulation disorders, which are found in 22–55% of hospitalized patients (32). In the present study, the high D-dimer serum level in the recruited patients reflects the underlying COVID-19 induced-coagulation disorders.

Specifically looking at the core of the present context, which was to address whether metformin improved clinical and laboratory findings in COVID-19 patients when compared with non-metformin treated COVID-19 patients, a lower percentage of unilateral paralysis in metformin-treated patients when compared to non-metformin treated patients was shown. Similar findings were also disclosed by Mima et al. (33), who showed that metformin therapy in T2DM patients with AIS reduces both acute neurological deficit and severity. Further, the findings obtained here illustrate that metformin therapy in T2DM patients with COVID-19 and AIS was associated with a better cardiometabolic profile and inflammatory cytokines levels compared with non-metformin-treated patients, mostly attributed to their anti-inflammatory effects and ability to improve the IR. Similarly, Kow and his colleague (34) found that metformin therapy is associated with a reduction in the mortality rate in hospitalized COVID-19 patients with T2DM due to its anti-inflammatory and antiviral effects. Indeed, it has been stated that the anti-inflammatory effect of metformin is through the activation of the AMPK pathway that inhibits the mTOR signaling and NF-kB pathway with subsequent suppression of IL-6 and TNF-α with the activation of anti-inflammatory IL-10. These changes triggered by metformin are also able to attenuate the cytokine production from activated macrophage and glial cells during severe COVID-19 and AIS-induced neuroinflammation (35). Zeng et al. (36) confirmed that metformin has a neuroprotective effect via the inhibition of apoptosis and oxidative stress in AIS and thus can be viewed as a promising preventive agent against ischemic-reperfusion in AIS. However, biomarkers of oxidative stress and cellular activity of NF-kB and/or AMPK pathways were not evaluated in the present study in relation to metformin therapy (37, 38).

Furthermore, the NLR was higher in T2DM patients with AIS and COVID-19 compared to the healthy controls due to neutrophil activation and lymphopenia. In fact, both AIS and COVID-19 are associated with high NLR, with a high NLR in AIS being linked to poor neurological outcomes and risk of intra-cerebral hemorrhage (39), while high NLR in COVID-19 patients is regarded as an independent risk factor for COVID-19 hospitilization and associated with a high mortality rate (40). Dermirdal et al. (41) also found that metformin reduces NLR in patients with T2DM. From this, it can be seen that neutrophils play a fundamental role in the inflammatory responses to ischemia/reperfusion injury by releasing oxidants, proteases, toll like receptors (TLRs) activation, and releasing inflammatory products (42). Despite these findings, metformin therapy in the present study was not associated with a reduction of NLR, probably due to the small sample size or the dosage amount of metformin not being sufficient to overcome neutrophil recruitment. Soraya et al. (43) confirmed the dose-dependent effect of metformin in the reduction of neutrophil activation and recruitment.

Of note, metformin therapy has been linked to a lower SRS compared to non-metformin treated patients due to its neuroprotective effects (39). Metformin pre-treatment with metformin in T2DM patients has also been associated with reduced neurological severity during AIS development (44). The potential neuroprotective effect of metformin in AIS has also been related to an AMPK dependent-inhibition of the NFκB pathway, cytokine activation, and associated blood brain barrier disruption with significant amelioration of neuronal glucose-oxygen consumption (33). Venna et al. (45) confirmed that metformin therapy improves post-stroke recovery through the modulation of AMPK signaling. Nevertheless, post-stroke outcomes were not addressed in the present study.

Undeniably, the present study illustrated that metformin therapy was linked to a lower ALI in COVID-19 patients, as revealed by a recent report (45). However, although Do et al. (46) revealed an insignificant effect of metformin on the amelioration of ALI in T2DM patients with COVID-19, Wu et al. (47) confirmed that metformin therapy may alleviate the endotoxemia-induced ALI through restoration of lung AMPK dependent inhibition of mTOR signaling. Thus, metformin therapy in T2DM patients with COVID-19 and AIS leads to dual protective effects on both the lung and brain.

On the other hand, metformin has potential antiviral effects against different viruses through the activation of the AMPK pathway (47). Recently, different studies have confirmed the antiviral effect of metformin against SARS-CoV-2 replication (48). The anti-SARS-CoV-2 activity of metformin is related to different mechanisms, namely the AMPK activation by metformin that leads to phosphorylation of the ACE2 receptor at Ser-680, where the interaction, stabilization, and conformational changes of ACE2 occur. These changes prolong the half-life of bound and soluble ACE2 and become less sensitive for SARS-CoV-2 binding (35). Also, the protective role of metformin against ALI in COVID-19 patients seems to be related to lung ACE2 up-regulation, exerting both anti-inflammatory and anti-apoptotic effects. Additionally, up-regulated ACE2 prevents the deleterious effect of high Ang II level in COVID-19-induced pneumonia (49). Dalan (50) found that metformin mitigates ALI in COVID-19-induced pneumonia through the inhibition of neutrophil migration and chemotaxis with a mast cell stabilizing effect.

Therefore, despite the small sample size, this study discloses for the first time the dual protective effect of metformin against COVID-19-induced pneumonia, ALI, and AIS through amelioration of hyper-inflammation and underlying cardiometabolic disturbances.

This study also has several limitations, the first one related to the small sample size and the second one related to the retrospective nature, so that long-term outcomes were not determined. In addition, gender differences were not evaluated since most recruited patients were males. The level of soluble ACE2 serum level was also not determined. Nevertheless, due to its strengths, this should be considered a preliminary study to trigger future large-scale prospective studies to confirm the link between ALI and AIS in DM patients with COVID-19.

## Conclusion

Metformin therapy in T2DM patients was associated with a lower risk of AIS during COVID-19. Further studies are needed to observe the link between AIS in COVID-19 diabetic patients under metformin therapy. However, we cannot draw any ultimate conclusions from our observation due to the small sample size. Therefore, we hypothesized that metformin therapy may attenuate and treat Covid-19 and associated AIS and ALI, prospective, randomized controlled studies are recommended in this regard.

## Data Availability Statement

The original contributions presented in the study are included in the article/supplementary material, further inquiries can be directed to the corresponding author/s.

## Ethics Statement

The studies involving human participants were reviewed and approved by Clinical Research and Ethical Committee Board, College of Medicine, Al-Mustansiryia University. The patients/participants provided their written informed consent to participate in this study.

## Author Contributions

All authors listed have made a substantial, direct and intellectual contribution to the work, and approved it for publication.

## Conflict of Interest

The authors declare that the research was conducted in the absence of any commercial or financial relationships that could be construed as a potential conflict of interest.

## References

[1] Yang L Liu S Liu J Zhang Z Wan X Huang B. COVID-19: immunopathogenesis and Immunotherapeutics. Signal Transduc Target Ther. (2020) 5:1–8. 10.1038/s41392-020-00243-2 PMC7381863 32712629

[2] Al-Kuraishy HM Al-Naimi MS Lungnier CM Al-Gareeb AI. Macrolides and COVID-19: an optimum premise. Biomed Biotechnol Res J. (2020) 4:189. 10.4103/bbrj.bbrj_103_20

[3] Ntaios G Michel P Georgiopoulos G Guo Y Li W Xiong J. Characteristics and outcomes in patients with COVID-19 and acute ischemic stroke: the global COVID-19 stroke registry. Stroke. (2020) 51:e254–8. 10.1161/STROKEAHA.120.031208 32787707 PMC7359900

[4] Belani P Schefflein J Kihira S Rigney B Delman BN Mahmoudi K. COVID-19 is an independent risk factor for acute ischemic stroke. Am J Neuroradiol. (2020) 41:1361–4. 10.3174/ajnr.A6650 32586968 PMC7658882

[5] Tan YK Goh C Leow AS Tambyah PA Ang A Yap ES. COVID-19 and ischemic stroke: a systematic review and meta-summary of the literature. J Thromb Thromb. (2020) 50:587–95. 10.1007/s11239-020-02228-y 32661757 PMC7358286

[6] Morelli N Rota E Terracciano C Immovilli P Spallazzi M Colombi D. The baffling case of ischemic stroke disappearance from the casualty department in the COVID-19 era. Eur Neurol. (2020) 14:1. 10.1159/000507666 32289789 PMC7179532

[7] Ye G Gao Q Qi P Wang J Hu S Chen K. The role of diabetes mellitus on the thrombus composition in patients with acute ischemic stroke. Interv Neuroradiol. (2020) 26:329–36. 10.1177/1591019919896940 31924102 PMC7254620

[8] Lee SH Jang MU Kim Y Park SY Kim C Kim YJ. Effect of prestroke glycemic variability estimated glycated albumin on stroke severity and infarct volume in diabetic patients presenting with acute ischemic stroke. Front Endocrinol. (2020) 11:230. 10.3389/fendo.2020.00230 32373074 PMC7186307

[9] Zhu L She ZG Cheng X Qin JJ Zhang XJ Cai J. Association of blood glucose control and outcomes in patients with COVID-19 and pre-existing type 2 diabetes. Cell Metab. (2020) 31:1068–77.e3. 10.1016/j.cmet.2020.04.021 32369736 PMC7252168

[10] Al-Kuraishy HM Sami OM Hussain NR Al-Gareeb AI. Metformin and/or vildagliptin mitigate type II diabetes mellitus induced-oxidative stress: the intriguing effect. J Adv Pharm Technol Res. (2020) 11:142. 10.4103/japtr.JAPTR_18_20 33102198 PMC7574736

[11] Sharif-Askari NS Sharif-Askari FS Mdkhana B Al Heialy S Ratemi E Alghamdi M. Effect of common medications on the expression of SARS-CoV-2 entry receptors in liver tissue. Arch Toxicol. (2020) 94:4037–41. 10.1007/s00204-020-02869-1 32808185 PMC7430937

[12] Gao Y Liu T Zhong W Liu R Zhou H Huang W. Risk of metformin in patients with type 2 diabetes with COVID-19: a preliminary retrospective report. Clin Transl Sci. (2020) 13:1055–9. 10.1111/cts.12897 32955785 PMC7537216

[13] Scheen AJ. Metformin and COVID-19: from cellular mechanisms to reduced mortality. Diabetes Metab. (2020) 46:423–6. 10.1016/j.diabet.2020.07.006 32750451 PMC7395819

[14] Al-kuraishy HM Al-Gareeb AI Al-Buhadilly AK. Rosuvastatin improves vaspin serum levels in obese patients with acute coronary syndrome. Diseases. (2018) 6:9. 10.3390/diseases6010009 29337850 PMC5871955

[15] Easton JD Saver JL Albers GW Alberts MJ Chaturvedi S Feldmann E. Definition and evaluation of transient ischemic attack: a scientific statement for healthcare professionals from the American Heart Association/American Stroke Association Stroke Council; Council on Cardiovascular Surgery and Anesthesia; Council on Cardiovascular Radiology and Intervention; Council on Cardiovascular Nursing; and the Interdisciplinary Council on Peripheral Vascular Disease: the American Academy of Neurology affirms the value of this statement as an educational tool for neurologists. Stroke. (2009) 40:2276–93. 10.1161/STROKEAHA.108.192218 19423857

[16] Ma LL Li BH Jin YH Deng T Ren XQ Zeng XT. Developments, evolution, and implications of national diagnostic criteria for COVID-19 in china. Front Med. (2020) 7:242. 10.3389/fmed.2020.00242 32574333 PMC7243174

[17] Francone M Iafrate F Masci GM Coco S Cilia F Manganaro L. Chest CT score in COVID-19 patients: correlation with disease severity and short-term prognosis. Eur Radiol. (2020) 30:6808–17. 10.1007/s00330-020-07033-y 32623505 PMC7334627

[18] Al-Kuraishy HM Al-Gareeb AI. Effect of orlistat alone or in combination with Garcinia cambogia on visceral adiposity index in obese patients. J Intercult Ethnopharmacol. (2016) 5:408. 10.5455/jice.20160815080732 27757272 PMC5061485

[19] Al-Kuraishy HM Al-Gareeb AI Naji MT. Brain natriuretic peptide in patients with acute ischemic stroke: role of statins. Biomed Biotechnol Res J. (2020) 4:239. 10.4103/bbrj.bbrj_44_20

[20] Zaki N Alashwal H Ibrahim S. Association of hypertension, diabetes, stroke, cancer, kidney disease, and high-cholesterol with COVID-19 disease severity and fatality: a systematic review. Diabetes Metab Syndr Clin Res Rev. (2020) 14:1133–42. 10.1016/j.dsx.2020.07.005 32663789 PMC7340589

[21] Saban-Ruiz J Ly-Pen D. COVID-19: a personalized cardiometabolic approach for reducing complications and costs. The role of aging beyond topics. J Nutr Health Aging. (2020) 4:550–9. 10.1007/s12603-020-1385-5 32510105 PMC7217344

[22] Qin C Zhou L Hu Z Yang S Zhang S Chen M. Clinical characteristics and outcomes of COVID-19 patients with a history of stroke in Wuhan, China. Stroke. (2020) 51:2219–23. 10.1161/STROKEAHA.120.030365 32466735 PMC7282412

[23] Yaghi S Ishida K Torres J Mac Grory B Raz E Humbert K. SARS2-CoV-2 and stroke in a New York healthcare system. Stroke. (2020) 51:e179. 10.1161/STROKEAHA.120.030335 32432996 PMC7258764

[24] Spence JD De Freitas GR Pettigrew LC Ay H Liebeskind DS Kase CS. Mechanisms of stroke in COVID-19. Cerebrovasc Dis. (2020) 49:451–8. 10.1159/000509581 32690850 PMC7445374

[25] Huertas A Montani D Savale L Pichon J Tu L Parent F. Endothelial cell dysfunction: a major player in SARS-CoV-2 infection (COVID-19)? Eur Respir J. (2020) 56:2001634 10.1183/13993003.01634-2020 32554538 PMC7301835

[26] Vas P Hopkins D Feher M Rubino F B Whyte M. Diabetes, obesity and COVID-19: a complex interplay. Diabetes Obes Metab. (2020) 22:1892–6. 10.1111/dom.14134 32627299 PMC7362013

[27] Whetton AD Preston GW Abubeker S Geifman N. Proteomics and informatics for understanding phases and identifying biomarkers in COVID-19 disease. J Proteome Res. (2020) 19:4219–32. 10.1021/acs.jproteome.0c00326 32657586 PMC7384384

[28] Shahjouei S Naderi S Li J Khan A Chaudhary D Farahmand G. Risk of stroke in hospitalized SARS-CoV-2 infected patients: a multinational study. EBioMedicine. (2020) 59:102939. 10.1016/j.ebiom.2020.102939 32818804 PMC7429203

[29] Babapoor-Farrokhran S Rasekhi RT Gill D Babapoor S Amanullah A. Arrhythmia in COVID-19. SN Compreh Clin Med. (2020) 14:1–6. 10.1007/s42399-020-00454-2 PMC7426193 32838188

[30] Wu L O'Kane AM Peng H Bi Y Motriuk-Smith D Ren J. SARS-CoV-2 and cardiovascular complications: from molecular mechanisms to pharmaceutical management. Biochem Pharmacol. (2020) 178:114114. 10.1016/j.bcp.2020.114114 32579957 PMC7306106

[31] Drago F Gozzo L Li L Stella A Cosmi B. Use of enoxaparin to counteract COVID-19 infection and reduce thromboembolic venous complications: a review of the current evidence. Front Pharmacol. (2020) 11:1469. 10.3389/fphar.2020.579886 33041824 PMC7525088

[32] Paolisso P Bergamaschi L D'Angelo EC Donati F Giannella M Tedeschi S. Preliminary experience with low molecular weight heparin strategy in COVID-19 patients. Front Pharmacol. (2020) 11:1124. 10.3389/fphar.2020.01124 32848743 PMC7424043

[33] Mima Y Kuwashiro T Yasaka M Tsurusaki Y Nakamura A Wakugawa Y. Impact of metformin on the severity and outcomes of acute ischemic stroke in patients with type 2 diabetes mellitus. J Stroke Cerebrovasc Dis. (2016) 25:436–46. 10.1016/j.jstrokecerebrovasdis.2015.10.016 26725260

[34] Kow CS Hasan SS. Mortality risk with preadmission metformin use in patients with COVID-19 and diabetes: a meta-analysis. J Med Virol. (2020) 93:695–7. 10.1002/jmv.26498 32902868

[35] Chen X Guo H Qiu L Zhang C Deng Q Leng Q. Immunomodulatory and antiviral activity of metformin and its potential implications in treating coronavirus disease 2019 and lung injury. Front Immunol. (2020) 11:2056. 10.3389/fimmu.2020.02056 32973814 PMC7461864

[36] Zeng J Zhu L Liu J Zhu T Xie Z Sun X. Metformin protects against oxidative stress injury induced by ischemia/reperfusion via regulation of the lncRNA-H19/miR-148a-3p/Rock2 axis. Oxid Med Cell Longev. (2019) 2019:8768327. 10.1155/2019/8768327 31934270 PMC6942897

[37] Liu Y Tang G Li Y Wang Y Chen X Gu X. Metformin attenuates blood-brain barrier disruption in mice following middle cerebral artery occlusion. J Neuroinflammation. (2014) 11:1–2. 10.1186/s12974-014-0177-4 25315906 PMC4201919

[38] Zhang J Dong J Martin M He M Gongol B Marin TL. AMP-activated protein kinase phosphorylation of angiotensin-converting enzyme 2 in endothelium mitigates pulmonary hypertension. Am J Respir Crit Care Med. (2018) 198:509–20. 10.1164/rccm.201712-2570OC 29570986 PMC6118028

[39] Zhang J Ren Q Song Y He M Zeng Y Liu Z. Prognostic role of neutrophil–lymphocyte ratio in patients with acute ischemic stroke. Medicine. (2017) 96:e8624. 10.1097/MD.0000000000008624 29137097 PMC5690790

[40] Liu Y Du X Chen J Jin Y Peng L Wang HH. Neutrophil-to-lymphocyte ratio as an independent risk factor for mortality in hospitalized patients with COVID-19. J Infect. (2020) 81:e6–12. 10.1016/j.jinf.2020.04.002 32283162 PMC7195072

[41] Demirdal T Sen P. The significance of neutrophil-lymphocyte ratio, platelet-lymphocyte ratio and lymphocyte-monocyte ratio in predicting peripheral arterial disease, peripheral neuropathy, osteomyelitis and amputation in diabetic foot infection. Diabetes Res Clin Pract. (2018) 144:118–25. 10.1016/j.diabres.2018.08.009 30176260

[42] Xue J Huang W Chen X Li Q Cai Z Yu T. Neutrophil-to-lymphocyte ratio is a prognostic marker in acute ischemic stroke. J Stroke Cerebrovasc Dis. (2017) 26:650–7. 10.1016/j.jstrokecerebrovasdis.2016.11.010 27955949

[43] Soraya H Rameshrad M Mokarizadeh A Garjani A. Metformin attenuates myocardial remodeling and neutrophil recruitment after myocardial infarction in rat. BioImpacts BI. (2015) 5:3. 10.15171/bi.2015.02 25901291 PMC4401166

[44] Deng T Zheng YR Hou WW Yuan Y Shen Z Wu XL. Pre-stroke metformin treatment is neuroprotective involving AMPK reduction. Neurochem Res. (2016) 41:2719–27. 10.1007/s11064-016-1988-8 27350579

[45] Venna VR Li J Hammond MD Mancini NS McCullough LD. Chronic metformin treatment improves post-stroke angiogenesis and recovery after experimental stroke. Eur J Neurosci. (2014) 39:2129–38. 10.1111/ejn.12556 24649970 PMC4061245

[46] Do JY Kim SW Park JW Cho KH Kang SH. Is there an association between metformin use and clinical outcomes in diabetes patients with COVID-19? Diabetes Metab. (2020) 9:1953. 10.1016/j.diabet.2020.10.006 33160030 PMC7640920

[47] Wu K Tian R Huang J Yang Y Dai J Jiang R. Metformin alleviated endotoxemia-induced acute lung injury via restoring AMPK-dependent suppression of mTOR. Chem Biol Interact. (2018) 291:1–6. 10.1016/j.cbi.2018.05.018 29859833

[48] Honda M Shirasaki T Terashima T Kawaguchi K Nakamura M Oishi N. Hepatitis B virus (HBV) core-related antigen during nucleos (t) ide analog therapy is related to intra-hepatic HBV replication and development of hepatocellular carcinoma. J Infect Dis. (2016) 213:1096–106. 10.1093/infdis/jiv572 26621908

[49] Ursini F Ciaffi J Landini MP Meliconi R. COVID-19 and diabetes: is metformin a friend or foe? Diabetes Res Clin Pract. (2020) 164:108167. 10.1016/j.diabres.2020.108167 32339534 PMC7195096

[50] Dalan R. Metformin, neutrophils and COVID-19 infection. Diabetes Res Clin Pract. (2020) 164:108230. 10.1016/j.diabres.2020.108230 32446796 PMC7242188

